# Prevalence of, and factors associated with health supplement use in Dubai, United Arab Emirates: a population-based cross-sectional study

**DOI:** 10.1186/s12906-019-2593-6

**Published:** 2019-07-12

**Authors:** Naseem Mohammed Abdulla, Faisal Aziz, Iain Blair, Michal Grivna, Balazs Adam, Tom Loney

**Affiliations:** 1 Health and Safety Department, Dubai Municipality, Dubai, United Arab Emirates; 2 Institute of Public Health, College of Medicine and Health Sciences, United Arab Emirates University, Al Ain, United Arab Emirates; 3 Division of Occupational Health, Department of Preventive Medicine, Faculty of Public Health, University of Debrecen, Debrecen, Hungary; 4 College of Medicine, Mohammed Bin Rashid University of Medicine and Health Sciences, PO Box 505055, Dubai, United Arab Emirates

**Keywords:** Attitudes, Dietary supplement, Dubai, Drug-related side effects and adverse reactions, Health knowledge, Practice, United Arab Emirates

## Abstract

**Background:**

Health supplement (HS) products that are available in the Emirate of Dubai (United Arab Emirates; UAE) contain chemicals that may adversely affect human health. This study aimed to investigate the prevalence of, and factors associated with HS consumption, knowledge, related adverse events, and reporting practices of adverse events amongst the general population in Dubai, UAE.

**Methods:**

A cross-sectional household telephone survey using a computer-assisted questionnaire was conducted amongst a random representative sample (*n* = 1203) of the Dubai population that assessed HS use and knowledge. Dependent variables were supplement use and reports of adverse events while independent variables included socio-demographic factors, knowledge, attitudes, and practice. Logistic regression analysis was performed to identify factors independently associated with HS use.

**Results:**

Among the 1203 participants in this study, 455 (37.8%) reported ever using HS. Amongst ever-users, reasons for use were to improve health (66.1%), for bodybuilding (9.9%), disease prevention (6.8%), and weight management (5.3%). The majority of users purchased their HS from pharmacies (88.4%) or were prescribed HS (46.6%). Vitamins were the most commonly used HS (87.9%) followed by minerals (10.5%) and sports nutrition products (10.5%). Only 2.9% of users experienced an adverse event associated with HS use which all resolved when the HS was discontinued. Only three of those affected reported the incident. Multivariate logistic regression analysis revealed that HS use was independently associated with female gender (adjusted odds ratio [AOR]; 3.26, 95% confidence interval [CI]: 2.26–4.70), higher income (AOR 2.41, 95% CI: 1.20–4.83), being a past-smoker (AOR 2.39, 95% CI: 1.27–4.48), having an allergy (AOR 1.75, 95% CI: 1.14–2.66), more frequent doctor visits (AOR 1.86, 95% CI: 1.02–3.39), taking prescribed medications (AOR 1.47, 95% CI: 1.04–2.06), and knowledge about HS (AOR 3.91, 95% CI: 2.26–6.76).

**Conclusions:**

Our study provides the first population-based estimates of HS use and HS-related adverse events in the Gulf region. Adverse events associated with HS are infrequent and this may be due to the well-developed regulatory framework in Dubai and the high level of knowledge amongst consumers who mainly consume vitamins and minerals on the advice of pharmacists or healthcare professionals.

**Electronic supplementary material:**

The online version of this article (10.1186/s12906-019-2593-6) contains supplementary material, which is available to authorized users.

## Introduction

In the Emirate of Dubai (United Arab Emirates; UAE) dietary and herbal supplements are combined under a common definition of “health supplements” (HS) [1]. This definition includes a wide variety of products ingested in many forms (e.g. capsules, tablets, powders, and liquids) containing vitamins, minerals, amino/fatty acids, herbs, and other dietary components to meet or improve essential nutritional requirements [1]. Such supplements may contain one or more different chemical ingredients which can cause adverse events by their chemical reactions within the human body. In this paper, we adapted the World Health Organization definition of an adverse event as any unfavourable health occurrence in a participant that was deemed to be associated with administration of a specific HS [2].

HS products such as minerals and vitamins are widely available over-the-counter and are often used without advice from a healthcare professional for indications including weight reduction and energy enhancement [3]. As HS products have a wide range of possible actions, their effectiveness and safety for human consumption is of concern since harmful adverse events have been reported following the use of some types of HS products [4]. For example, *Ginkgo biloba* has been implicated in the occurrence of epileptic seizures, and chronic use of zinc may result in anemia [5–7]. Other adverse events include allergic reactions, heavy metal poisoning, and reactions to adulterants or contaminants [8]. When problems with HS occur, the presenting clinical features may provide clues about the offending agents and a careful approach to management is required including symptomatic and supportive care, and in some cases resuscitation [8]. HS products with clear pharmacological effects or toxic constituents can be inherently poisonous, and physicians should anticipate problems if they encounter patients using these products [9]. Also, potentially hazardous interactions between HS products and some medicines have been reported including synergistic effects, poisoning, or inactivation leading to a reduced therapeutic effect [4]. For example, St. John’s wort (*Hypericum perforatum*), a plant derived product that is used to treat mild and moderate depression, can induce liver enzymes and therefore has the potential to interact with many narrow therapeutic range medicines that are metabolized by the liver such as anti-depressants [10]. Other substances such as garlic, ginger, and *Ginkgo biloba* can increase the risk of bleeding when administered with anticoagulants [11]. In many countries, spontaneous reporting or vigilance systems are the main mechanisms for detecting safety issues associated with HS use [12]. If suspected adverse events associated with HS use are not identified either through direct patient reporting or indirect reporting by healthcare professionals, the recognition of important safety issues may be delayed or even missed completely [13, 14].

Globally, HS are widely consumed and a recent systematic review reported that the prevalence of regular HS use ranged from 22 to 53% amongst the general population in Canada (Female (F) and Male (M) combined 40%), France (F 46%; M 24%), Germany (F 39%; M 29%), Korea (F 32%; M 22%), Sweden (F 33%; M 22%), the United Kingdom (F 46%; M 35%), and the United States (F 53%; M 44%) [15]. At present, there is a lack of population-based estimates for countries in the Middle East region. However, there is an emerging body of research suggesting similar or higher HS use amongst specific sub-groups or non-representative samples of the general population in these countries: 71.4% of adult Kuwaiti nationals [16], 35.0% of Lebanese adults [17], 89.7% of pregnant Omani women [18], 32.7% of University students in Qatar [19], and 76.6% of female college students in Saudi Arabia reported regular HS use [20]. Currently, there are limited data and information on HS use and related adverse events in the Dubai population which is one of the seven emirates of the United Arab Emirates (UAE). Moreover, there is no surveillance or reporting system for adverse events resulting from HS use in Dubai. Therefore, the primary aim of this study was to investigate the prevalence of, and factors associated with HS consumption, knowledge, related adverse events and reporting practices amongst the population in Dubai, UAE.

## Methods

This section has been written in accordance with the Strengthening the Reporting of Observational Studies in Epidemiology (STROBE) guidelines [21].

### Study design and setting

A cross-sectional household telephone survey using a computer-assisted questionnaire was conducted amongst a random representative sample (*n* = 1203) of the Dubai population aged 16 years or older from 01 September to 31 December 2015. Dubai is the second largest of the UAE’s seven Emirates with a population of approximately 2.45 million in 2015 [22]. It is a highly developed modern city on the shores of the Arabian Gulf with very good education and healthcare infrastructure.

### Participants and study size

All Dubai residents aged 16 years and above were eligible to participate in the study. All households in Dubai are required to have an active landline and/or mobile telephone number registered to their residential address. In 2015, there were 1,349,101 landlines and 5,696,569 mobile telephone active lines in Dubai [22]. Dubai Statistics Center provided the sampling frame which was a list of all residential addresses and telephone numbers in Dubai grouped into six geographical areas based on demographic and socio-economic considerations. Based on previously published studies, it was assumed that the prevalence of HS use in the target population would be approximately 30% [3, 23–25]. Estimating the true prevalence with a precision of ± 3% would require a sample of 1067 and this was increased by 12% to allow for non-response so that the final sample size was 1200.

### Data collection

Two hundred addresses were randomly selected from each of the six geographical locations. For each address, up to 20 mobile phone numbers were available and one of these numbers was randomly sampled. A trained researcher telephoned the selected number to explain the purpose of the study and to ensure the respondent was aged 16 years or over. In the event that the respondent was underage or otherwise ineligible, the researcher randomly selected another number from that residential address. At the end of the study approximately 6200 mobile numbers had been contacted in this way (estimated 20% response rate). The study protocol was approved by the Social Sciences Research Ethics Committee of the United Arab Emirates University. All the participants provided verbal informed consent.

### Questionnaire measures

A computer-assisted telephone-administered questionnaire was developed to obtain relevant information related to socio-demographic and lifestyle factors (independent variables) and HS use and experience of HS-related adverse events (dependent variables). The 48-item questionnaire was adapted from previously used surveys and reviews on HS use and adverse events [7, 8, 19, 23–25]. The questionnaire consisted of six sections, and it contained both open- and close-ended questions (see Additional file 1). The first section *‘Demographic Data’* included 10 items collecting information on age, gender, marital status, nationality, occupation, health insurance coverage, monthly income, education, and self-reported body mass (kg) and height (cm). Body mass index (BMI) was calculated as body mass in kilograms divided by height in metres squared (kg/m^2^). Section two *‘Health and Lifestyle’* was composed of five items collecting data on allergies, frequency of doctor visits within the past 12 months, diagnosis of chronic medical conditions, prescription drug use in the past month, and smoking status. Section three *‘Health Supplements Consumption’* included 12 questions on knowledge of HS and HS use including type, frequency, duration, form, ingredients, and reasons for taking and/or discontinuation of HS, source of purchase, and number of HS products ever used. Section four *“Information about Health Supplement Products”* contained seven items on source of HS advice, HS prescription, and usefulness and adherence to HS label advice/information. Section five *‘Adverse Events to Health Supplement Consumption’* contained 10 questions on frequency, type, severity, timing of adverse events, and seeking medical treatment. Section six *‘Reporting Adverse Events’* was composed of four items collecting data on the reporting of ‘ever’ experiencing an adverse event and perceptions on the usefulness of an adverse event surveillance system.

### Quantitative variables

Nationality was collapsed into six categories: Emirati, Middle East/North Africa, South Asia, East Asia/Pacific/Central Asia/Europe, Africa, and Latin America/Caribbean/Western Europe/North America/Australia. Monthly income in Emirati dirhams (AED) was categorized into AED < 5000, AED 5000- < 10000, AED 10000–20000, and AED > 20000 (USD 1.00 ≈ AED 3.67). Education was categorized as less than high school, high school, graduate, or postgraduate. Smoking status was categorized into non-smoker, past smoker, current occasional smoker, and current regular smoker. Frequency of visiting a doctor in the last 12 months was categorized as *‘Did not visit a doctor in the last 12 months’*, *‘Less than monthly’, and ‘1-3 times a month/At least once a week’*. A binary (Yes/No) variable was created for knowledge of HS from the question *‘Do you know what health supplements are?’* (see Additional file 1).

### Bias

The following strategies were used to minimize possible bias: (i) in an attempt to minimize selection bias and maximize the possibility of recruiting a representative sample, the study used stratified (based on geographical area) random sampling from a sampling frame provided by the Dubai Statistics Center that contained a list of all residential addresses and telephone numbers in Dubai; (ii) the telephone-administered questionnaire was piloted on a random sample (*n* = 134; 48.4% Emirati) of Arabic and English speaking participants. No changes were made to the questionnaire following the pilot study; however, pilot sample participants were not included in the final analysis; (iii) interviewers fluent in Arabic, English, and other commonly spoken languages in Dubai (e.g. Urdu) were hired and completed a training course on the specific data collection procedure; (iv) data collection was supervised and used computer-assisted telephone interviewing to ensure standardization of procedures across interviewers; and (v) although data collection was supervised, telephone interviews were repeated on a random sub-sample of 40 participants by an independent researcher for interview verification and quality assurance purposes; specifically, the independent researcher called the telephone number to verify that the interview had been conducted.

#### Statistical analysis

Data were exported from the computer application as a Microsoft Excel spreadsheet and analyses were conducted using STATA version 14.2. Descriptive statistics were used to describe the demographic characteristics of the sample using frequencies (percentages) or means (standard deviation) as appropriate. Difference between subgroups of the sample (age, gender, educational status, nationality) were tested using a Chi-square test for categorical variables and ANOVA for continuous variables. Simple logistic regression analysis was performed to assess the association between HS use (outcome variable) and selected correlates (independent variables). The variables having *p* value < 0.10 were included in a stepwise logistic regression model using backward elimination to identify the independent factors associated with HS use. The confidence interval of 95% and *p* value ≤0.05 were used to determine statistical significance.

## Results

**Fig. 1:**
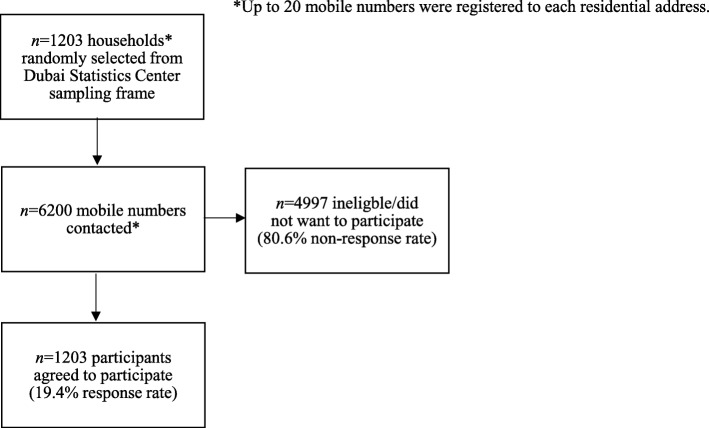
Flow of participant recruitment for the study

A total of 6200 mobile numbers were called from the Dubai Statistics Center sampling frame and 1203 agreed to participate (19.4% response rate; Fig. 1). Among the 1203 participants in this study, 37.8% (*n* = 455) reported ever using health supplements and of these only 2.9% (*n* = 13) had experienced an adverse event (Fig. 1). Participants were predominantly male and married (Table 1). South Asians constituted the largest ethnic group in the study, followed by Middle East/ North Africa, UAE and Western Europe/ North America/ Australia. There were smaller numbers of participants from other countries.

**Table 1:** Characteristics of participants, cross-sectional study of HS use and HS-related adverse events, Dubai, 2015 (*n* = 1203)

Variables	*n*/Mean	%/SD
Mean Age (years)	39.2	±9.1
Gender		
Male	1002	83.3
Female	201	16.7
Marital Status		
Married	1039	86.4
Single	150	12.5
Divorced	9	0.7
Widow	5	0.4
Nationality		
Emirati	142	11.8
Middle East/North Africa	301	25.0
South Asia	579	48.1
Western Europe/North America/Australia	94	7.8
Other countries	87	7.3
Occupation		
Employed	1123	93.3
Unemployed	60	5.0
Student	9	0.7
Retired	11	0.9
Income, AED (~USD)^a^		
AED < 5000 (~USD < 1300)	164	13.6
AED 5000- < 10000 (~USD 1300- < 2700)	499	41.5
AED 10000–20000 (~USD 2700–5450)	319	26.5
AED > 20000 (~USD > 5450)	221	18.4
Education		
< High school	76	6.3
High school	139	11.5
Diploma	68	5.7
Higher Diploma	46	3.8
Bachelor	586	48.7
Master	269	22.4
PhD	19	1.6
Health insurance coverage	1028	85.5
Height (cm)	171.3	±8.9
Body mass (kg)	78.7	±15.3
Mean (± SD) Body Mass Index (kg/m^2^)	26.8	±4.4
Body Mass Index category		
Normal (< 25 kg/m^2^)	431	35.8
Overweight (25–29.9 kg/m^2^)	546	45.4
Obese (≥30 kg/m^2^)	226	18.8
Smoking status		
Non-smoker	862	71.7
Past smoker	50	4.2
Current occasional	108	8.9
Current regular	183	15.2
Any allergy	115	9.6
Drug allergy	21	1.7
Aerosol & perfume allergy	23	1.9
Contact allergy	7	0.6
Dust allergy	22	1.8
Others	26	2.2
Diseases^b^		
Diabetes Mellitus	69	5.7
High cholesterol levels	31	2.6
Cardiovascular disease	5	0.4
Cancer	1	0.1

Note. ^a^Based on USD 1.00 ≈ AED 3.67. ^b^Respondents could choose more than one answer

The majority of respondents were employed and 41.5% (*n* = 499) had a monthly income in the range of AED 5,000- < 10,000 (USD 1.00 ≈ AED 3.67), 319 participants (26.5%) earned between AED 10,000-20,000, 221 participants (18.4%) had an income higher than AED 20,000 while only 164 participants (13.6%) earned less than AED 5,000.

Educational attainment varied across the sample: nearly half of participants (48.7%) had completed undergraduate degrees, 22.4% postgraduate education, and 11.5% had completed high school education (Table 1). The majority (85.4%) of participants had health insurance coverage. Mean (±SD) body mass index was 26.8 ± 4.4 kg/m^2^ and nearly two-thirds (64.2%) of participants were classified as overweight or obese according to their body mass index (Table 1). Nearly three-quarters (71.7%) of the sample were non-smokers, 15.2% were current regular smokers, 8.9% were current occasional smokers and 4.2% were past smokers. Of the total participants, 9.6% had an allergy, mainly to aerosols or perfume. Less than 10% reported medical conditions, mainly diabetes mellitus (5.7%) and hypercholesterolemia (2.6%).

### Health supplement use

**Fig. 2:**
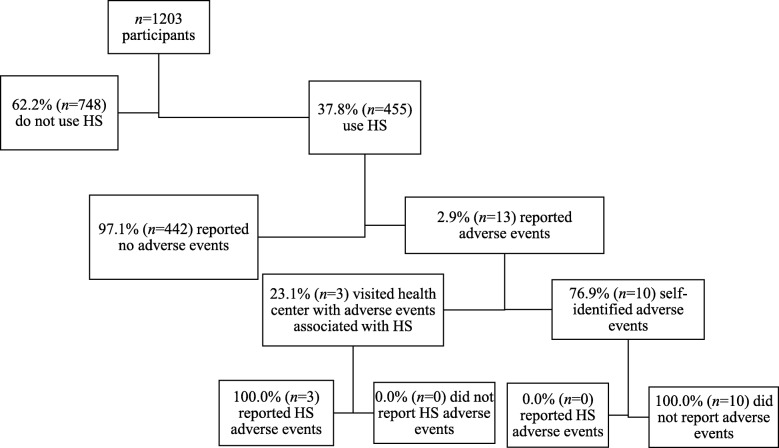
Flow of participants with adverse events

Amongst all respondents, 85.5% answered affirmatively that they knew about HS and 37.8% had used HS at least once of whom 26.4% were currently using HS (Table 2). Amongst ever-users, the main reasons for use were to improve health (66.1%) and for bodybuilding (9.9%; Table 2). Amongst ever-users, 41.5% had used HS for between 1 and 12 months, 36.3% had used HS for 1–5 years, and 9.9% had used HS for more than five years (Table 2). The majority of HS users (63.3%) were daily users and consumed 1–2 different types of HS (85.3%; Table 2). The majority (88.3%) of HS users purchased their HS from pharmacies (Table 2)(Fig. 2).

**Table 2:** Use of health supplements among participants in Dubai, 2015 (*n* = 1203)

Variables	N	n (%)
HS use	1203	
Ever used (Including current and past users)		455 (37.8)
Current		317 (26.4)
Past		138 (11.5)
Never		748 (62.2)
Reasons for using HS^a^	455	
To improve health		301 (66.1)
Body building (Male only)		45 (9.9)
Disease prevention		31 (6.8)
Diet supplementation		27 (5.9)
Weight management		24 (5.3)
Energy		18 (4.0)
Pregnancy (Female only)		11 (2.4)
Immunity booster		8 (1.8)
Cold Prevention		8 (1.8)
Digestive		6 (1.3)
Aging		5 (1.1)
High cholesterol		5 (1.1)
Anaemia		4 (0.9)
High blood pressure		3 (0.7)
Other		8 (1.7)
Duration of HS use, ever users	455	
Less than a month		52 (11.4)
Month		189 (41.5)
1–5 years		165 (36.3)
More than 5 years		45 (9.9)
Do not know		4 (0.9)
Frequency of HS use	455	
Seasonally		17 (3.7)
< 1 a month		9 (2.0)
1–3 times a month		22 (4.8)
1–4 times a week		116 (25.5)
Daily		288 (63.3)
Do not know		3 (0.7)
Number of HS	455	
1–2 supplements		388 (85.3)
3–5 supplements		59 (13.0)
6–10 supplements		6 (1.3)
> 10 supplements		2 (0.4)
Purchasing of HS^a^	455	
Pharmacy		402 (88.3)
Clinic		45 (9.9)
Nutrition shop		29 (6.7)
Gym		12 (2.6)
Super market		5 (1.1)
Other		9 (2.0)

Note. ^a^Respondents could choose more than one answer

### Information and advice to use HS

Among the 455 participants who had ever used HS, 46.6% were prescribed HS and 44.8% decided for themselves to use HS (Additional file 2: Table S1). Smaller numbers of respondents were advised by other healthcare professionals, advised by friends or relatives, or persuaded to use HS by advertising (Additional file 2: Table S1). The majority of ever users (60.2%) obtained information about HS from pharmacies (Additional file 2: Table S1). Other sources of information included the internet (31.9%), physicians (28.4%) and relatives or friends (8.6%; Additional file 2: Table S1). Most ever users reported that they found the HS labelling information very informative (78.0%), particularly with respect to ingredients and possible adverse events and that they always followed the instructions (Additional file 2: Table S1).

### HS forms and ingredients

Vitamins were the most commonly used HS (87.9%) followed by minerals (10.5%) and sport nutrition products (10.5%; Additional file 3: Table S2). The most widely used HS form was tablet form (85.5%). The commonest ingredients reported were Vitamins A, D, and E (Additional file 3: Table S2). Very few respondents did not know the ingredients of their HS (Additional file 3: Table S2).

### HS adverse events

Of the 455 ever users of HS, only 2.9% had experienced an adverse event associated with HS use (Additional file 4: Table S3). These were generally isolated adverse events, of mild or moderate severity which resolved when the HS was discontinued. The reported presentations included abdominal pain, dermatitis, diarrhoea, constipation, and urticaria (Additional file 4: Table S3). Amongst those reporting adverse events, nine participants reported taking vitamin products and five participants reported taking sports nutrition products (Additional file 4: Table S3). However, only five participants that experienced an adverse event “blamed” a specific product as the *cause* of the adverse event (Additional file 4: Table S3). Vitamin products were responsible in four cases while in the fifth a “slimming tea” product was blamed (Additional file 4: Table S3). Only three of those affected reported the incident (Additional file 4: Table S3).

### Correlates of HS use

Additional file 5: Table S4 summarizes the bivariate analysis in which HS use (dependent variable) is tabulated against independent variables. There was no association with age but females were significantly more likely to be HS users. Single and divorced/widowed respondents were more likely to report HS use as were persons of Latin America/ Caribbean/ Western Europe/ North America/ Australia origin. Participants of South Asian origin were less likely to report HS use. Employed persons and those with incomes over AED 10,000 per month were more likely to be HS users compared to those who were unemployed or on lower salaries. Educational attainment was significantly associated with HS with those educated to higher diploma level and above being more likely to report HS use compared to those of lower educational attainment. Those with health insurance were more likely to report HS use, as were those with an allergy, those who reported visiting their doctor and those taking medicines. Body mass index, smoking, and self-reports of medical conditions were not associated with HS use. Finally, those who answered affirmatively to the question *“Do you know what health supplements are?”* were significantly more likely to report HS use.

The association between HS use as an outcome variable and selected population characteristics as independent variables is summarized in Additional file 6: Table S5. There was a positive association between HS use and female gender, higher income, higher educational level, having health insurance, being a past-smoker, having an allergy, more frequent doctor visits, taking prescribed medications, and HS knowledge (Additional file 6: Table S5). There was a negative association between HS use and being married and Emirati, Middle East/North Africa or South Asian nationality. After adjustment in the multivariate model, the positive association with female gender, higher income, being a past-smoker, having an allergy, more frequent doctor visits, taking prescribed medications and HS knowledge and the negative association with South Asian nationality and Emirati nationality remained (Table 3).

**Table 3:** Adjusted odds ratios for HS use in Dubai, 2015 (*n* = 1203)

Variables	AOR (95%CI)
Sex	
Male	1
Female	3.26 (2.26–4.70)**
Nationality	
Emirati	0.55 (0.30–1.00)*
Middle East/North Africa	0.66 (0.39–1.12)
South Asia	0.51 (0.28–0.93)*
East Asia/Pacific/Central Asia/Europe	1.16 (0.53–2.52)
Africa	0.50 (0.21–1.22)
Latin America/Caribbean/Western Europe/North America/Australia	1
Income, AED (~USD)^a^	
AED < 5000 (~USD < 1300)	1
AED 5000- < 10000 (~USD 1300- < 2700)	1.18 (0.71–1.98)
AED 10000–20000 (~USD 2700–5450)	1.83 (0.98–3.41)
AED > 20000 (~USD > 5450)	2.41 (1.20–4.83)*
Allergy	
Yes	1.75 (1.14–2.66)*
No	1
Smoking status	
Non-smoker	1
Past smoker	2.39 (1.27–4.48)**
Current occasional smoker	0.85 (0.52–1.36)
Current regular smoker	0.93 (0.64–1.36)
Visited to a doctor in last 12 months	
Did not visit doctor in last 12 months	1
Less than monthly	1.37 (0.96–1.94)
1–3 times a month/At least once a week	1.86 (1.02–3.39)*
Prescribed Medicines	
Yes	1.47 (1.04–2.06)*
No	1
Knowledge of HS	
Yes	3.91 (2.26–6.76)**
No	1

Note. Stepwise regression method using backward elimination was used to identify significant correlates (*p* < 0.10) of HS use. *p < 0.05 **p < 0.01. ^a^Based on USD 1.00 ≈ AED 3.67

## Discussion

Our study is the first to report population-based estimates of HS use and HS-related adverse events in a major city in the Gulf region. In our sample, more than a third (38%) of respondents reported using HS and the majority (88%) consumed vitamins with health improvement as the predominant (66%) reason for HS use. Despite widespread use of HS amongst the Dubai population, adverse events associated with HS use were relatively rare (< 5%). Interestingly, we found that HS use was independently positively associated with female gender, higher income, being a past smoker, having an allergy, more frequent doctor visits, taking prescribed medications, and knowledge about HS. Conversely, there was a negative association between HS use and being Emirati or South Asian.

Previous population-based surveys in the United States have shown that approximately half of the population regularly consume HS [25]. The National Health and Nutrition Examination Survey (NHANES) reported that the percentage of the United States population who used at least one HS increased from 42% in 1988–1994 to 53% in 2003–2006 [25]. A recent serial cross-sectional study using nationally representative NHANES data (*n* = 37,958) collected between 1999 and 2012 found that the use of HS remained stable between 1999 and 2012, with 52% of adults reporting use of any supplements in 2011–2012 [26]. Currently, there is a lack of population-based surveys on HS use in the Middle East. However, our population-based prevalence estimates are similar to other studies in the Middle East that examined HS use amongst specific sub-groups or non-representative samples of the general population. A cross-sectional study in Kuwait used multi-stage stratified clustered sampling to recruit a representative sample of Kuwaiti citizens (*N* = 1173; 56.4% female; 90.2% response rate) in 2010–11 and revealed that 71.4% of the sample used HS [16]. The study excluded expatriates that constitute approximately two-thirds of the Kuwaiti population and the estimates cannot be extrapolated to the general population. A similar cross-sectional study in Lebanon recruited a convenient sample of ambulating Lebanese adults (*N* = 726; 59.8% female; response rate 93.0%) attending randomly selected community pharmacies from six Lebanese governorates [17]. Consenting participants completed a face-to-face interview that collected data on herbal product and dietary supplement consumption patterns. Nearly one-quarter (23%) of Lebanese adults reported to be currently consuming vitamins and/or mineral supplements and 18% herbal supplements [1]. Two other cross-sectional studies in the Gulf region recruited a random sample of females (*N* = 534; aged 19–26 years) attending one University in Saudi Arabia [20] and a random sample of males and females (*N* = 419; 59.2% male; response rate 82.4%) attending two private universities in Qatar [19]. Over three-quarters (76.6%) of female university students in Saudi Arabia reported dietary supplement use and higher levels of education and physical activity were associated with health supplement use compared to non-users [19]. In Qatar, nearly half (49.6%) of private university students had ‘ever’ used supplements, 32.7% reported use in the past six months (current users), and 58.5% deemed supplements to be safer than medicine [19]. It was difficult to assess overall knowledge and awareness of HS in our sample but nearly all respondents knew what HS were and amongst users about a half had been prescribed their HS and 60% had received information about HS from a pharmacist, suggesting a reasonable level of awareness.

Of the users, only 3% reported experiencing adverse events and most of these were not serious. The low level of adverse events experienced by HS users may account for the finding that amongst all respondents, 46% were unsure if there would be any benefits from establishing a reporting system for HS-related adverse events. That said, most participants expressed the view that a reporting system would be beneficial.The published literature identifies a high risk of adverse events associated with HS use, especially herbal supplements as they have a greater chance to have been contaminated by, for example, heavy metals and pesticide residues [27–29]. Previous research suggests that up to 10% of HS users experience an associated adverse events [30–32]. It was somewhat surprising that the prevalence of adverse events in the present study was only 3%. A retrospective analysis of clinical drug poisoning cases (*n* = 1474) from 2011 to 2016 in the city of Jeddah in Saudi Arabia reported that only 3.6% of poisoning caused by drug overdose were due to vitamins and the majority of these were accidental (82.7%) and in the 0–4 year old age group (84.6%) [33]. One reason for the lower prevalence reported in Dubai may be because vitamin products were the most common type of HS rather than more problematic preparations containing herbs. A second reason could be the closer monitoring and control of HS availability in the Dubai market-place by the authorities compared with other countries with higher reported rates. A third reason may be that consumers are more knowledgeable and are using HS under pharmacist supervision. A final reason could be the low prevalence of sport nutrition consumption (~ 11%) in our sample as these products have been associated with a higher prevalence of adverse events. In 2014, a cross-sectional study using a random sample (*n* = 1708) of United States Royal Navy and Marine Corps personnel reported that 29% of HS users (dietary/nutritional supplement) reported at least one adverse event in the previous six month period [34].

The finding that adverse events, albeit uncommon, are usually unreported is noteworthy. Currently, there is no adverse events monitoring system in Dubai. Consideration should be given to establishing such a system and raising awareness amongst consumers of the importance of reporting adverse events. This will permit ongoing surveillance of HS-related adverse events and ensure that any future increases can be promptly recognized.

In our study we found strong and significant associations between use of HS and female gender, higher income, history of allergy, being a past-smoker, more frequent to doctors and taking of prescribed medication. These associations may not be surprising as use of HS may be part of a spectrum of health behaviors where consumers are seeking treatment or substitutes in combination with allopathic medicine and prescribed pharmaceuticals. Less than half of our sample had been prescribed HS by a physician. Self-reported diagnosis of diabetes mellitus (36.2%) and cardiovascular disease (45.2%) was relatively high amongst HS users. The concomitant use of prescribed allopathic medicine with over-the-counter HS creates a challenge for physicians as HS can interfere with the pharmacokinetics and pharmacodynamics of prescribed medicine causing reduced efficacy and possibly adverse events [3]. In 2015, a cross-sectional survey on the knowledge and attitudes towards complementary and alternative medicine amongst senior (clinical phase) medical students (*n* = 242) from one university in Saudi Arabia reported very low awareness of commonly used herbs such as St. John’s Wort, *Echinacea*, and *Gingko biloba* [33]. In view of these findings, healthcare professionals need to possess sufficient knowledge about HS to provide accurate advice to their patients [35].

Clearly, HS can be expensive and the cost may be prohibitive to members of the Dubai population without health insurance and/or with lower incomes. We found that HS is strongly associated with female gender. This finding is well known and is thought to be explained by women’s greater health consciousness, although women also have higher utilization of allopathic health services [36]. We found lower HS use amongst Emiratis and those of South Asian origin. Ethnic, nationality and cultural differences in HS use have been reported in the literature but results are variable and this should be an area for future enquiry [37].

### Strengths & Limitations

The main strength of this study was the recruitment of a large random representative sample using trained multi-lingual researchers to conduct the telephone-based computer-assisted interview. The large representative sample permits comparisons with other studies and ensures more precise estimates with sufficient power to detect associations between HS use and potential explanatory variables. The major limitation of this study is the cross-sectional design which does not allow for the determination of temporality or causality between independent and dependent variables. Another limitation is that the sample may not be completely representative of the overall Dubai population as we used a minimum age requirement of 16 years which slightly reduces the generalizability of the findings. There is limited data available on the age, gender, and nationality structure of the Dubai population to compare the representativeness of our sample. However, estimates from 2016 indicate that our sample with a minimum age of 16 years was slightly older (mean age 39 years versus median age for the population of 30 years), contained a higher proportion of males (83% versus 70%), and a marginally higher proportion of Emiratis (11.8% versus 8.6%) [22]. There are logistical and legal challenges to obtaining sampling frames in the UAE to draw representative samples for population-based cross-sectional studies [22, 38]. Our sample was slightly older due to our minimum age requirement and included more males than the most recently reported population structure of Dubai (sample 83% versus population 70%) [22]. The higher proportion (> 80%) of males in our sample may be related to the family and social hierarchical structure in the UAE national population whereby the head of household is usually male and therefore, the telephone number and billing is usually linked to a male head of household. Finally, information on HS use and adverse events was based on self-reports and so may be subject to concerns about validity and information bias.

### Implications for Practice & Further Research

Our population-based household telephone survey reported that one third of the general population in Dubai aged ≥16 years use HS, mainly vitamins, on a regular basis. Moreover, approximately half of users had been prescribed HS by a physician and 60% received HS information from a pharmacist. In view of these findings and the high levels of self-reported chronic disease in our sample, the curricula of medical and pharmacology schools in the UAE needs to cover the possible interactions and adverse events associated with the concomitant use of prescribed medicine with commonly used over-the-counter HS. Despite the relatively low prevalence (< 5%) of adverse events associated with HS use, Dubai may benefit from an online, mobile app, or telephone-based reporting system as this would raise awareness amongst consumers and allow the long-term monitoring of adverse events in Dubai. As with any cross-sectional design, it is not possible to draw conclusions about the temporal or causal nature of the association between proposed independent and dependent variables. Future studies utilizing more robust longitudinal cohort designs would allow researchers to relate different sociodemographic variables with HS use, or even HS use with specific adverse events. Such studies may want to consider focusing on higher-risk individuals including children, pregnant women, and older adults with co-morbidities.

## Conclusion

For the first time in the Gulf region, we have estimated HS use and HS-related adverse events in a representative sample of the general population that included both UAE nationals and expatriates. Levels of HS use are similar to those reported in other studies but the HS-related adverse events are infrequent. The current well-developed regulatory framework for HS availability and use in Dubai may be the reason for this reassuring situation. Nevertheless, we recommend that an adverse events monitoring system is established to complement the steps that have already been taken to ensure the continued safe use of HS by the people of Dubai.

## Additional files

Additional file 1: Health Supplement Use in Dubai Telephone Questionnaire. (PDF 579 kb)

Additional file 2: **Table S1** Information and advice to use HS amongst ever users of HS in Dubai, 2015 (*n* = 455). (DOCX 16 kb)

Additional file 3: **Table S2** Forms and ingredients of HS reported by ever users of HS in Dubai, 2015 (*n* = 455). (DOCX 16 kb)

Additional file 4: **Table S3** Adverse events from HS use reported by ever users of HS in Dubai, 2015 (*n* = 455). (DOCX 17 kb)

Additional file 5: **Table S4** Correlates of HS use, cross-sectional study of HS use and HS-related adverse events, Dubai, 2015 (*n* = 1203). (DOCX 20 kb)

Additional file 6: **Table S5** Crude odds ratios for HS use, cross-sectional study of HS use and HS-related adverse events, Dubai, 2015 (*n* = 1203). (DOCX 20 kb)

## Data Availability

The datasets generated and/or analysed during the current study are not publicly available as this was not included in the original ethics application. However, the dataset is available from the corresponding author on reasonable request and approval from the local research ethics committee. Additional data is available in the Supplementary Tables.

## Abbreviations

AE: Adverse Events
AED: Emirati Dirham
AOR: Adjusted Odds Ratio
CI: Confidence Interval
F: Female
HS: Health Supplements
M: Male
SD: Standard Deviation
UAE: United Arab Emirates
USD: United States Dollar
