# Supplementary material for: Prevalence of, and factors associated with health supplement use in Dubai, United Arab Emirates: a population-based cross-sectional study
Source: BMC Complement Altern Med. 2019 Jul 12;19:172. doi: 10.1186/s12906-019-2593-6 (PMC6624985; doi:10.1186/s12906-019-2593-6)
Supplement: Supplementary file 1 — Health Supplement Use in Dubai Telephone Questionnaire. (PDF 579 kb) [file 12906_2019_2593_MOESM1_ESM.pdf]

Health Supplement Use in Dubai Telephone Questionnaire

**Section A- Demographic Data**				
**Age** (years/months)				
**Gender**	Male ◻		Female ◻	
**Marital Status**	Married ◻	Single ◻	Divorced ◻	Widowed ◻
**Nationality**	UAE National ◻	Non-UAE National ◻ (Specify …………………...)		
**Occupation**	Student ◻	Employed ◻	Unemployed ◻	Retired ◻
**Health Insurance Coverage**	Yes ◻		No ◻	
**Income**	<5000 AED ◻	5000-<10000 AED ◻	10000-20000 AED ◻	>20000AED ◻
**Education**	Less than High School ◻	High School ◻	Graduate ◻	Postgraduate ◻
**Weight (kg)**				
**Height (cm)**				

**Section B- Health and Lifestyle**					
**1**	**Do you have any allergy?**				
	Yes ◻ *(if yes, please choose from below options)*		No ◻	Don't know ◻	
	Food ◻	Drug ◻	Aerosol ◻	Contact ◻	Other ◻ Specify___________
**2**	**How frequently have you visited a doctor in the past 12 months?**				
	At least once a week ◻	1-3 times a month ◻	Less than monthly ◻	Never ◻	
**3**	**Have you ever been diagnosed with any chronic medical condition?** *(you can choose more than one answer)*				
	Respiratory disease	No ◻	Yes ◻	Specify	(…)
	Skin disorder	No ◻	Yes ◻	Specify	(…)
	Disease of the digestive system	No ◻	Yes ◻	Specify	(…)
	Diabetes	No ◻	Yes ◻	Specify	(…)
	Cardiovascular disease	No ◻	Yes ◻	Specify	(…)
	Cancer	No ◻	Yes ◻	Specify	(…)
	Other	No ◻	Yes ◻	Specify	(…)
**4**	**Have you taken prescription drugs in the past month?**				
	Yes ◻ Specify (………)		No ◻	Don't know ◻	
**5**	**Do you smoke?**				
	Every day ◻	Occasionally ◻	In the past ◻	Never ◻	

Section C- Health Supplements Consumption	
**1**	**Do you know what health supplements are?**<br>Yes □ No □
**2**	**Have you ever used health supplement?** *(if your answer is "Currently", please proceed to question C4, if your answer is "Never", please proceed to the last question of the questionnaire)*<br>Currently □ In the past □ Never □
**3**	**Why did you discontinue using any health supplements?** *(you can choose more than one answer)*<br>Allergic reactions □ Serious skin disorders □ Cost □ Others □ Specify (…)
**4**	**For how long have you been using / had you used health supplement?**<br>Less than a month □ More than a month but less than a year □ 1-5 years □ > 5 years □ Don't know □
**5**	**How frequently do/did you use health supplement?**<br>Daily or almost daily □ 1-4 times a week □ 1-3 times a month □ Rarer than monthly through the year □ Seasonally □ Don't know □
**6**	**Which categories of health supplements do /did you use?** *(you can choose more than one answer)*<br>Vitamins □ Minerals □ Herbal □ Sports nutrition □ Energy drink □<br>Dietetic food □ Others □ Specify (…………………)
**7**	**What is the form of the used product(s)?** *(you can choose more than one answer)*<br>Tablet □ Capsule □ Wafers □ Powder □ Gel □ Chews/ Gummy □ Drops □ Caplet □ Chewable tablets □<br>Granules □ Drink □ Spray □ Lozenges □ Soft gels □ Vegicaps □ Gel caps □ Liquid □ Don't know □

**8**	Which health supplement ingredient(s) do/did you use? (you can choose more than one answer)

Alfalfa	◻	Amino Acids	◻	Bee Pollen	◻
Bilberry/Eyebright Combination	◻	Caffeine, Multicomponent	◻	Calcium	◻
Calcium & Magnesium	◻	Calcium & Vitamin	◻	Cayenne Pepper	◻
Chondroitin	◻	Chromium (Chromium Picolinate)	◻	Cimicifuga Racemosa	◻
Conjugated Linolenic Acid	◻	Creatine	◻	Damiana Folia	◻
Don't Know	◻	Echinacea	◻	Ephedra	◻
Fish Oils	◻	Folate (Folic Acid)	◻	Fructus Cynosbati	◻
Garlic	◻	Gentian, Multi-Component	◻	Ginger	◻
Ginkgo Biloba	◻	Glandular Extract, Multicomponent	◻	Glucosamine	◻
Grape Seed Extract	◻	Guarana	◻	Herbal Caffeine, Alone	◻
Iron (Ferrous Xxxate)	◻	Kelp	◻	L-Carnitine	◻
L-Cysteine	◻	Lecithin	◻	Licorice	◻
L-Methionine	◻	Lutein	◻	Lycopene	◻
Lysine	◻	Magnesium	◻	Melatonin	◻
Methylsulfonyl Methane	◻	Morinda Citrifolia (Noni)	◻	Oxymatrine	◻
Panax Ginseng	◻	Parsley	◻	Potassium	◻
Pygeum Africanum	◻	Royal Jelly	◻	Saw Palmetto	◻
Saw Palmetto (Topical)	◻	Selenium	◻	Siberian Ginseng	◻
Spirulina, Multicomponent	◻	St. John's Wort	◻	St. John's Wort, Multicomponent	◻
Tryptophan	◻	Vitamin B6	◻	Vitamin B12	◻
Vitamin C (With Or Without Rose Hips)	◻	Vitamin D	◻	Vitamin E	◻
Vitamin E, Multicomponent	◻	Vitamins A & D	◻	Yohimbe, Alone	◻
Yohimbe, Multicomponent	◻	Zinc (Zinc Gluconate)	◻	Others Specify (…….)	◻

**9**	**For what reason do/did you take health supplements?** *(you can choose more than one answer)*

Body building		Control aging	□	Control anemia	
Control blood pressure	□	Control cholesterol level	□	Detoxify	□
Digestive	□	Energy booster	□	Hormone therapy	□
Immune booster	□	Improve overall health	□	Insomnia	□
Memory improvement	□	Menopausal	□	Mental alertness	□
Mood alteration	□	Organ health Specify (……………..)	□	Pregnancy	□
Prevent colds	□	Prevent health problems Specify (……………..)	□	Supplement my diet	□
Weight management	□	Others Specify (……..)	□		

**10**	**Where do/did you purchase health supplement(s)?** *(you can choose more than one answer)*<br>Pharmacy □ Clinic □ Gym □ Nutrition shops □<br>Supermarket □ Other □ Specify (……..….)
**11**	**How many health supplement products have you ever used?**<br>1-2 □ 3-5 □ 6-10 □ >10 □
**12**	**Enter the full name of health supplement(s) you have used, including brand name.**<br>Supplement name(s) (……………………….……………….)<br>Don't know □

Section D- Information about Health Supplement Products	
**1**	**Who advised you to take health supplements?** *(you can choose more than one answer)*<br>Self-recommendation □ Friends/ Relatives □ Advertisement □ Internet □<br>By prescription □ Health care personnel (nurse, etc.) □ Other □ Specify (………...)
**2**	**How many times have health supplements been prescribed for you by your health care practitioner?**<br>Once □ Twice □ Several times □<br>Never □
**3**	**Where do you seek health supplements product information?** *(you can choose more than one answer)*<br>Pharmacy □ Physician □ Producer helpline □ Internet □<br>Government call center □ Relatives / Friends □ Other □<br>Specify (..........................)
**4**	**Do you find sufficient information on the label of health supplement products?**<br>Very informative □ Somewhat informative □ Not informative □<br>Don’t read the label □
**5**	**Do you think nutrition information on health supplement products is useful?**<br>Yes □ No □
**6**	**Which label information concerns you?** *(you can choose more than one answer)*<br>Ingredients □ Indication □ Prescribed dosages □ Adverse reactions □ Product durability □<br>Dietary sources of nutrients □ Claims □ Precautions □ Dosing instructions □<br>None □
**7**	**Do you follow recommended label information?**<br>Always □ Often □ Sometimes □ Never □

Section E- Adverse Events Related to Health Supplement Consumption	
**1**	**Have you ever experienced any adverse event related to health supplement use?** *(if no, please proceed to the last question of the questionnaire)*<br>Yes □ No □
**2**	Which adverse event of health supplement use have you ever experienced? (you can choose more than one answer)<br>Abdominal pain □ Anorexia □ Anxiety □ Chest pain □ Convulsions □<br>Dermatitis □ Diarrhea □ Dizziness □ Dyspnea □ Edema □<br>Fatigue □ Hair loss □ Headache □ Hypertension □ Hypotension □<br>Muscle cramping □ Muscle pain □ Nausea □ Palpitations □ Pyrexia □<br>Sedation □ Tingling □ Urticaria □ Vomiting □ Other □ Specify….........
**3**	**What was the severity of the adverse events?** *(you can choose more than one answer)*<br>Mild □ Moderate □ Severe □ Life-threatening □

**4**	**How frequently have you encountered adverse events due to health supplement consumption?**<br><br>Once □ Occasionally □ Frequently □
**5**	**What was the onset time of adverse events after consuming health supplement?** *(you can choose more than one answer)*<br>Less than 1 hour □ 1 hour to 1 day □ More than 1 day □
**6**	**How was the relation between health supplement consumption and the adverse event confirmed?** *(you can choose more than one answer)*<br>Discontinued use ceased the effect □ Not confirmed/personal opinion □<br>Physician opinion □ Medical diagnosis without lab confirmation □<br>Clinical test □
**7**	**Which of the health supplement(s) you have used was suspected/confirmed to cause the adverse event(s)?**<br>Supplement name(s) (…………………….) Don't know □
**8**	**When visiting your health care practitioner for any reason, has he/she ever asked you about your health supplement consumption?**<br>Yes □ No □
**9**	**How did the adverse event(s) resolve?** *(you can choose more than one answer)*<br>**(***if you answered any but not "Hospitalization", please proceed to question F1)*<br>Discontinued use by personal decision □ Discontinued use by medical advice □<br>Medical treatment □ Hospitalization □ Resolved spontaneously □<br>Still persists □ Other □ Specify (………………..)
**10**	**How long have you been hospitalized due to the adverse event(s)?** *(you can choose more than one answer)*<br>Less than a day □ Few days □ More than a week □

**Section F- Reporting Adverse Events**	
**1**	**Have you ever informed your physician about your health supplement consumption?**<br>Yes □ No □
**2**	**Have you ever reported an adverse event related to health supplement consumption? (***if no, please proceed to the last question of the questionnaire)*<br>Yes □ No □
**3**	**Where did you report the adverse event(s)?** *(you can choose more than one answer)*<br>Pharmacy □ Physician □ Producer helpline □ Internet □ Government call center □<br>Hospital □ Clinic □ Police □ Others □ Specify (……….)
**4**	**What do you think about the establishment of a surveillance system of adverse events related to health supplement consumption?**<br>Definitely beneficial □ Somewhat beneficial □ Not sure □ Not beneficial □<br>Definitely not beneficial □
