# Supplementary material for: Prevalence of, and factors associated with health supplement use in Dubai, United Arab Emirates: a population-based cross-sectional study
Source: BMC Complement Altern Med. 2019 Jul 12;19:172. doi: 10.1186/s12906-019-2593-6 (PMC6624985; doi:10.1186/s12906-019-2593-6)
Supplement: Supplementary file 2 — Table S1 Information and advice to use HS amongst ever users of HS in Dubai, 2015 (n = 455). (DOCX 16 kb) [file 12906_2019_2593_MOESM2_ESM.docx]

Additional file 2: Table S1. Information and advice to use HS amongst ever users of HS in Dubai, 2015 (*n*=455)

| Variables | N | n (%) |
| --- | --- | --- |
| Who advised to take HS^‡^ | 455 |  |
| Self |  | 204 (44.8) |
| Friend/Relative |  | 35 (7.7) |
| Advertisement |  | 5 (1.1) |
| Internet |  | 30 (6.6) |
| Prescribed |  | 212 (46.6) |
| Health professional |  | 49 (10.8) |
| Other |  | 5 (1.1) |
| From where do you seek HS information^‡^ | 455 |  |
| Pharmacy |  | 274 (60.2) |
| Physician |  | 129 (28.4) |
| Product helpline |  | 10 (2.2) |
| Internet |  | 145 (31.9) |
| Relative/Friend |  | 39 (8.6) |
| Government centre |  | 0 (0.0) |
| Other |  | 4 (0.9) |
| Sufficient information on the label | 455 |  |
| Do not read the label |  | 28 (6.1) |
| Not informative |  | 4 (0.9) |
| Somewhat informative |  | 68 (14.9) |
| Very informative |  | 355 (78.0) |
| Type of label information of HS concerns | 455 |  |
| Ingredients of supplement |  | 375 (82.4) |
| Indications of supplement |  | 245 (53.9) |
| Dosage of supplement |  | 237 (52.1) |
| Adverse events of supplement |  | 291 (64.0) |
| Durability of supplement |  | 312 (68.6) |
| Dietary sources of supplement |  | 226 (49.8) |
| Claims of supplement |  | 199 (43.7) |
| Precautions of supplement |  | 233 (51.2) |
| Dosing instructions of supplement |  | 227 (49.9) |
| No information concerns |  | 41 (9.1) |
| Nutrition information on the label is useful |  | 431 (94.7) |
| Do you follow recommended label information? | 455 |  |
| Never |  | 22 (4.8) |
| Sometimes |  | 35 (7.7) |
| Often |  | 64 (14.1) |
| Always |  | 334 (73.4) |

Note. ^‡^Respondents could choose more than one answer. HS denotes Health Supplements.
