# Supplementary material for: Prevalence of, and factors associated with health supplement use in Dubai, United Arab Emirates: a population-based cross-sectional study
Source: BMC Complement Altern Med. 2019 Jul 12;19:172. doi: 10.1186/s12906-019-2593-6 (PMC6624985; doi:10.1186/s12906-019-2593-6)
Supplement: Supplementary file 3 — Table S2 Forms and ingredients of HS reported by ever users of HS in Dubai, 2015 (n = 455). (DOCX 16 kb) [file 12906_2019_2593_MOESM3_ESM.docx]

Additional file 3: Table S2. Forms and ingredients of HS reported by ever users of HS in Dubai, 2015 (*n*=455)

| Variables | N | n (%) |
| --- | --- | --- |
| Categories of HS^‡^ | 455 |  |
| Vitamins |  | 400 (87.9) |
| Minerals |  | 48 (10.5) |
| Herbal products |  | 9 (2.0) |
| Sports nutrition |  | 48 (10.5) |
| Energy drinks |  | 5 (1.1) |
| Dietetic food |  | 9 (2.0) |
| Miscellaneous |  | 12 (2.6) |
| Forms of HS^‡^ | 455 |  |
| Tablets |  | 389 (85.5) |
| Capsules |  | 53 (11.7) |
| Powder |  | 46 (10.1) |
| Drinks/Liquids/Caplets/Granules/Lozenges/Gels |  | 16 (3.5) |
| Ingredients of HS^‡^ | 455 |  |
| Vitamin D |  | 195 (42.9) |
| Vitamin E |  | 104 (22.9) |
| Vitamin A & D |  | 104 (22.9) |
| Calcium & Vitamins |  | 58 (12.7) |
| Vitamin B12 |  | 56 (12.3) |
| Calcium |  | 52 (11.4) |
| Vitamin C with/without rose |  | 48 (10.5) |
| Vitamin B6 |  | 45 (9.9) |
| Fish oil |  | 32 (7.0) |
| Amino acids |  | 27 (5.9) |
| Calcium & Magnesium |  | 24 (5.3) |
| Vitamin E multicomponent |  | 20 (4.4) |
| Zinc/zinc gluconate |  | 14 (3.1) |
| Magnesium |  | 7 (1.5) |
| Folate/Folic acid |  | 6 (1.3) |
| Potassium |  | 6 (1.3) |
| Carnitine |  | 5 (1.1) |
| Alfalfa |  | 1 (0.2) |
| Chondroitin |  | 2 (0.4) |
| Creatinine |  | 7 (1.5) |
| Other |  | 14 (3.1) |
| Do not know about ingredient |  | 14 (3.1) |

Note. ^‡^Respondents could choose more than one answer. HS denotes Health Supplements.
