# Supplementary material for: Prevalence of, and factors associated with health supplement use in Dubai, United Arab Emirates: a population-based cross-sectional study
Source: BMC Complement Altern Med. 2019 Jul 12;19:172. doi: 10.1186/s12906-019-2593-6 (PMC6624985; doi:10.1186/s12906-019-2593-6)
Supplement: Supplementary file 4 — Table S3 Adverse events from HS use reported by ever users of HS in Dubai, 2015 (n = 455). (DOCX 17 kb) [file 12906_2019_2593_MOESM4_ESM.docx]

Additional file 4: Table S3. Adverse events from HS use reported by ever users of HS in Dubai, 2015 (*n*=455)

| Variables | N | n (%) |
| --- | --- | --- |
| Adverse Events (AE) from HS | 13 | 13 (2.9) |
| Type of Adverse Events^‡^ |  |  |
| Abdominal pain |  | 1 (7.7) |
| Dermatitis |  | 2 (15.4) |
| Diarrhoea |  | 2 (15.4) |
| Constipation |  | 2 (15.4) |
| Urticaria |  | 3 (23.1) |
| Other |  | 3 (23.1) |
| Severity of AE from HS | 13 |  |
| Mild |  | 6 (46.1) |
| Moderate |  | 5 (38.5) |
| Severe |  | 2 (15.4) |
| Frequency of AE from HS | 13 |  |
| Once |  | 9 (69.2) |
| Occasionally |  | 2 (15.4) |
| Frequently |  | 2 (15.4) |
| Onset time of AE | 13 |  |
| <1 hour |  | 5 (38.5) |
| 1 hour – 1 day |  | 3 (23.1) |
| >1 day |  | 6 (46.1) |
| Relation between HS use and AE confirmed | 13 |  |
| Discontinued use ceased the effect |  | 10 (76.9) |
| Not confirmed/personal opinion |  | 2 (15.4) |
| Physician opinion |  | 1 (7.7) |
| Medical diagnosis without laboratory confirmation |  | 0 (0.0) |
| Clinic test |  | 0 (0.0) |
| Types of supplements taken by those reporting AE^‡^ | 13 |  |
| Vitamins |  | 9 (69.3) |
| Minerals |  | 0 (0.0) |
| Herbal products |  | 0 (0.0) |
| Sports nutrition |  | 5 (38.5) |
| Energy drinks |  | 0 (0.0) |
| Dietetic food |  | 1 (7.7) |
| Miscellaneous |  | 1 (7.7) |
| HS suspected/confirmed to cause AE | 13 |  |
| Vitamins |  | 4 (30.8) |
| Slimming tea |  | 1 (7.7) |
| How did the AE resolve? |  |  |
| Self-discontinuing the supplement |  | 10 (76.9) |
| Discontinuing the supplement after medical advise |  | 2 (15.4) |
| Treatment |  | 1 (7.7) |
| Ever reported AE | 13 | 3 (23.1) |
| Where did you report AE | 3 |  |
| Physician |  | 3 (100.0) |
| Benefits of establishing AE reporting system | 1203 |  |
| Definitely not beneficial |  | 15 (1.3) |
| Not beneficial |  | 11 (0.9) |
| Unsure |  | 550 (45.7) |
| Somewhat beneficial |  | 163 (13.5) |
| Definitely beneficial |  | 464 (38.6) |

Note. ^‡^Respondents could choose more than one answer. AE denotes Adverse Events; HS denotes Health Supplements.
