# Supplementary material for: Prevalence of, and factors associated with health supplement use in Dubai, United Arab Emirates: a population-based cross-sectional study
Source: BMC Complement Altern Med. 2019 Jul 12;19:172. doi: 10.1186/s12906-019-2593-6 (PMC6624985; doi:10.1186/s12906-019-2593-6)
Supplement: Supplementary file 5 — Table S4 Correlates of HS use, cross-sectional study of HS use and HS-related adverse events, Dubai, 2015 (n = 1203). (DOCX 20 kb) [file 12906_2019_2593_MOESM5_ESM.docx]

Additional file 5: Table S4. Correlates of HS use, cross-sectional study of HS use and HS-related adverse events, Dubai, 2015 (*n*=1203)

| Variables | All | Users (*n*=455) | Non-users  (*n*=748) |  |
| --- | --- | --- | --- | --- |
|  | N | n (%) | n (%) | P Value |
| Mean (± SD) Age (years) | 1203 | 38.9 ±9.0 | 39.4 ±9.1 | 0.307 |
| Sex |  |  |  |  |
| Male | 1002 | 322 (32.1) | 680 (66.9) | <0.001 |
| Female | 201 | 133 (66.2) | 68 (33.8) |  |
| Marital Status |  |  |  |  |
| Married | 1039 | 379 (36.5) | 660 (63.5) | 0.051 |
| Single | 150 | 69 (46.0) | 81 (54.0) |  |
| Divorced/Widow/Widower | 14 | 7 (50.0) | 7 (50.0) |  |
| Nationality |  |  |  |  |
| Emirati | 142 | 68 (47.9) | 74 (52.1) | <0.001 |
| Middle East/North Africa | 301 | 144 (47.8) | 157 (52.2) |  |
| South Asia | 579 | 141 (24.3) | 438 (75.6) |  |
| East Asia/Pacific/Central Asia/Europe | 53 | 28 (52.8) | 25 (47.2) |  |
| Africa | 32 | 14 (43.7) | 18 (56.3) |  |
| Latin America/Caribbean/Western Europe/North America/Australia | 96 | 60 (62.5) | 36 (37.5) |  |
| Occupation |  |  |  |  |
| Employed | 1123 | 413 (36.8) | 710 (63.2) | 0.003 |
| Unemployed | 60 | 35 (58.3) | 25 (41.7) |  |
| Student/Retired | 20 | 7 (35.0) | 13 (65.0) |  |
| Income, AED (~USD)^†^ |  |  |  |  |
| AED <5000 (~USD <1300) | 164 | 33 (20.1) | 131 (79.9) | <0.001 |
| AED 5000-<10000 (~USD 1300-<2700) | 499 | 149 (29.9) | 350 (70.1) |  |
| AED 10000-20000 (~USD 2700-5450) | 319 | 153 (48.0) | 166 (52.0) |  |
| AED >20000 (~USD >5450) | 221 | 120 (54.3) | 101 (45.7) |  |
| Education |  |  |  |  |
| < High school | 76 | 13 (17.1) | 63 (82.9) | <0.001 |
| High school | 139 | 39 (28.1) | 100 (71.9) |  |
| Diploma | 68 | 18 (26.5) | 50 (73.5) |  |
| Higher Diploma | 46 | 22 (47.8) | 24 (52.2) |  |
| Bachelor | 586 | 245 (41.8) | 341 (58.2) |  |
| Master/PhD | 288 | 118 (41.0) | 170 (59.0) |  |
| Health insurance coverage |  |  |  | 0.017 |
| Yes | 1028 | 403 (39.2) | 625 (60.8) |  |
| No | 175 | 52 (29.7) | 123 (70.3) |  |
| Body Mass Index-kg/m^2^ (mean ± SD) | 1203 | 26.9 ±4.5 | 26.7 ±4.3 | 0.348 |
| Body Mass Index categories |  |  |  |  |
| Normal (<25 kg/m^2^) | 431 | 165 (38.3) | 266 (61.7) | 0.496 |
| Overweight (25-29.9 kg/m^2^) | 546 | 198(36.3) | 348 (63.7) |  |
| Obese (≥30 kg/m^2^) | 226 | 92 (40.7) | 134 (59.3) |  |
| Smoking status |  |  |  |  |
| Non-smoker | 862 | 326 (37.8) | 536 (62.2) | 0.059 |
| Past smoker | 50 | 27 (54.0) | 23 (46.0) |  |
| Current occasional smoker | 108 | 34 (31.5) | 74 (68.5) |  |
| Current regular smoker | 183 | 68 (37.2) | 115 (62.8) |  |
| Any allergy |  |  |  | 0.006 |
| Yes | 115 | 57 (49.6) | 58 (50.4) |  |
| No | 1081 | 395 (36.5) | 686 (36.5) |  |
| Drug allergy |  |  |  | 0.129 |
| Yes | 20 | 13 (65.0) | 7 (35.0) |  |
| No | 95 | 44 (46.3) | 51 (53.7) |  |
| Aerosol and perfume allergy |  |  |  | 0.152 |
| Yes | 20 | 7 (35.0) | 13 (65.0) |  |
| No | 95 | 50 (52.6) | 45 (47.3) |  |
| Contact allergy |  |  |  | 0.252 |
| Yes | 7 | 2 (28.6) | 5 (71.4) |  |
| No | 108 | 55 (50.9) | 53 (49.1) |  |
| Dust allergy |  |  |  | 0.040 |
| Yes | 23 | 7 (30.4) | 16 (69.6) |  |
| No | 92 | 50 (54.3) | 42 (45.7) |  |
| Visited to a doctor in last 12 months |  |  |  |  |
| Did not visit doctor in last 12 months | 322 | 79 (24.5) | 243 (75.5) | <0.001 |
| Less than monthly | 806 | 342 (42.4) | 464 (57.6) |  |
| 1-3 times a month/ At least once a week | 75 | 34 (45.3) | 41 (54.7) |  |
| Diabetes Mellitus |  |  |  | 0.779 |
| Yes | 69 | 25 (36.2) | 44 (63.8) |  |
| No | 1134 | 430 (37.9) | 704 (62.1) |  |
| High cholesterol levels |  |  |  | 0.352 |
| Yes | 31 | 9 (29.0) | 22 (71.0) |  |
| No | 1172 | 446 (38.1) | 726 (61.9) |  |
| Cardiovascular disease |  |  |  | 0.393 |
| Yes | 31 | 14 (45.2) | 17 (54.8) |  |
| No | 1172 | 441 (37.6) | 731 (62.4) |  |
| Prescribed Medicines |  |  |  | <0.001 |
| Yes | 226 | 115 (50.9) | 111 (49.4) |  |
| No | 977 | 340 (34.8) | 637 (65.2) |  |
| Knowledge of HS |  |  |  | <0.001 |
| Yes | 1029 | 436 (42.4) | 593 (57.6) |  |
| No | 174 | 19 (10.9) | 155 (89.1) |  |

Note. Define as answering affirmatively to the question “Do you know what health supplements are?” ^†^Based on USD 1.00 ≈ AED 3.67. HS denotes Health Supplements; SD denotes Standard Deviation.
