# Supplementary material for: Prevalence of, and factors associated with health supplement use in Dubai, United Arab Emirates: a population-based cross-sectional study
Source: BMC Complement Altern Med. 2019 Jul 12;19:172. doi: 10.1186/s12906-019-2593-6 (PMC6624985; doi:10.1186/s12906-019-2593-6)
Supplement: Supplementary file 6 — Table S5 Crude odds ratios for HS use, cross-sectional study of HS use and HS-related adverse events, Dubai, 2015 (n = 1203). (DOCX 20 kb) [file 12906_2019_2593_MOESM6_ESM.docx]

Additional file 6: Table S5. Crude odds ratios for HS use, cross-sectional study of HS use and HS-related adverse events, Dubai, 2015 (*n*=1203)

| Variables | All | Users |  |
| --- | --- | --- | --- |
|  | N | n (%) | COR (95%CI) |
| Mean (± SD) Age (years) | 1203 | 38.9 ±9.0 | 0.99 (0.98-1.01) |
| Sex |  |  |  |
| Male | 1002 | 322 (32.1) | 1 |
| Female | 201 | 133 (66.2) | 4.13 (3.00-5.69)^**^ |
| Marital Status |  |  |  |
| Single | 150 | 69 (46.0) | 1 |
| Married | 1039 | 379 (36.5) | 0.67 (0.48-0.95)^*^ |
| Divorced/Widow/Widower | 14 | 7 (50.0) | 1.17 (0.39-3.51) |
| Nationality |  |  |  |
| Emirati | 142 | 68 (47.9) | 0.55 (0.33-0.93)^*^ |
| Middle East/North Africa | 301 | 144 (47.8) | 0.55 (0.34-0.88)^*^ |
| South Asia | 579 | 141 (24.3) | 0.19 (0.12-0.30)^**^ |
| East Asia/Pacific/Central Asia/Europe | 53 | 28 (52.8) | 0.67 (0.34-1.33) |
| Africa | 32 | 14 (43.7) | 0.47 (0.21-1.05) |
| Latin America/Caribbean/Western Europe/North America/Australia | 96 | 60 (62.5) | 1 |
| Occupation |  |  |  |
| Employed | 1123 | 413 (36.8) | 1.08 (0.43-2.73) |
| Unemployed | 60 | 35 (58.3) | 2.60 (0.91-7.44) |
| Student/Retired | 20 | 7 (35.0) | 1 |
| Income, AED (~USD)^†^ |  |  |  |
| AED <5000 (~USD <1300) | 164 | 33 (20.1) | 1 |
| AED 5000-<10000 (~USD 1300-<2700) | 499 | 149 (29.9) | 1.69 (1.10-2.59)^*^ |
| AED 10000-20000 (~USD 2700-5450) | 319 | 153 (48.0) | 3.66 (2.35-5.68)^**^ |
| AED >20000 (~USD >5450) | 221 | 120 (54.3) | 4.72 (2.96-7.50)^**^ |
| Education |  |  |  |
| <High school | 76 | 13 (17.1) | 1 |
| High school | 139 | 39 (28.1) | 1.89 (0.94-3.81) |
| Diploma | 68 | 18 (26.5) | 1.74 (0.78-3.90) |
| Higher Diploma | 46 | 22 (47.8) | 4.44 (1.93-10.20)^**^ |
| Bachelor | 586 | 245 (41.8) | 3.48 (1.87-6.47)^**^ |
| Master/PhD | 288 | 118 (41.0) | 3.36 (1.77-6.39)^**^ |
| Health insurance coverage |  |  |  |
| Yes | 1028 | 403 (39.2) | 1.53 (1.08-2.16)^*^ |
| No | 175 | 52 (29.7) | 1 |
| Body Mass Index categories |  |  |  |
| Normal (<25 kg/m^2^) | 431 | 165 (38.3) | 1 |
| Overweight (25-29.9 kg/m^2^) | 546 | 198(36.3) | 0.92 (0.71-1.19) |
| Obese (≥30 kg/m^2^) | 226 | 92 (40.7) | 1.11 (0.80-1.54) |
| Smoking status |  |  |  |
| Non-smoker | 862 | 326 (37.8) | 1 |
| Past smoker | 50 | 27 (54.0) | 1.93 () |
| Current occasional smoker | 108 | 34 (31.5) | 0.75 (0.49-1.16) |
| Current regular smoker | 183 | 68 (37.2) | 0.97 (0.70-1.35) |
| Any allergy |  |  |  |
| Yes | 115 | 57 (49.6) | 1.71 (1.16-2.51)^*^ |
| No | 1081 | 395 (36.5) | 1 |
| Visited to a doctor in last 12 months |  |  |  |
| Did not visit doctor in last 12 months | 322 | 79 (24.5) | 1 |
| Less than monthly | 806 | 342 (42.4) | 2.27 (1.70-3.03)^**^ |
| 1-3 times a month/ At least once a week | 75 | 34 (45.3) | 2.55 (1.51-4.29)^**^ |
| Diabetes Mellitus |  |  |  |
| Yes | 69 | 25 (36.2) | 0.93 (0.56-1.94) |
| No | 1134 | 430 (37.9) | 1 |
| High cholesterol levels |  |  |  |
| Yes | 31 | 9 (29.0) | 0.67 (0.30-1.46) |
| No | 1172 | 446 (38.1) | 1 |
| Cardiovascular disease |  |  |  |
| Yes | 31 | 14 (45.2) | 1.37 (0.66-2.80) |
| No | 1172 | 441 (37.6) | 1 |
| Prescribed Medicines |  |  |  |
| Yes | 226 | 115 (50.9) | 1.94 (1.45-2.60)^**^ |
| No | 977 | 340 (34.8) | 1 |
| Knowledge of HS |  |  |  |
| No | 174 | 19 (10.9) | 1 |
| Yes | 1029 | 436 (42.7) | 6.00 (3.67-9.81)** |

Note. **p*<.05 ***p*<.01. ^†^Based on USD 1.00 ≈ AED 3.67. COR denotes Crude Odds Ratio; HS denotes Health Supplements; SD denotes Standard Deviation.
